# Matrix Metalloproteases in Pancreatic Ductal Adenocarcinoma: Key Drivers of Disease Progression?

**DOI:** 10.3390/biology9040080

**Published:** 2020-04-18

**Authors:** Etienne J. Slapak, JanWillem Duitman, Cansu Tekin, Maarten F. Bijlsma, C. Arnold Spek

**Affiliations:** 1 Center of Experimental and Molecular Medicine, University of Amsterdam, Amsterdam UMC, 1105 AZ Amsterdam, The Netherlands; e.j.slapak@amsterdamumc.nl (E.J.S.); j.w.duitman@amsterdamumc.nl (J.D.); c.tekin@amsterdamumc.nl (C.T.); 2 Laboratory for Experimental Oncology and Radiobiology, Cancer Center Amsterdam, University of Amsterdam, Amsterdam UMC, 1105 AZ Amsterdam, The Netherlands; m.f.bijlsma@amsterdamumc.nl; 3 Oncode Institute, 1105 AZ Amsterdam, The Netherlands

**Keywords:** MMP, MMP2, MMP9, MMP7, MMP14, matrix metalloproteases, PDAC, pancreatic cancer

## Abstract

Pancreatic cancer is a dismal disorder that is histologically characterized by a dense fibrotic stroma around the tumor cells. As the extracellular matrix comprises the bulk of the stroma, matrix degrading proteases may play an important role in pancreatic cancer. It has been suggested that matrix metalloproteases are key drivers of both tumor growth and metastasis during pancreatic cancer progression. Based upon this notion, changes in matrix metalloprotease expression levels are often considered surrogate markers for pancreatic cancer progression and/or treatment response. Indeed, reduced matrix metalloprotease levels upon treatment (either pharmacological or due to genetic ablation) are considered as proof of the anti-tumorigenic potential of the mediator under study. In the current review, we aim to establish whether matrix metalloproteases indeed drive pancreatic cancer progression and whether decreased matrix metalloprotease levels in experimental settings are therefore indicative of treatment response. After a systematic review of the studies focusing on matrix metalloproteases in pancreatic cancer, we conclude that the available literature is not as convincing as expected and that, although individual matrix metalloproteases may contribute to pancreatic cancer growth and metastasis, this does not support the generalized notion that matrix metalloproteases drive pancreatic ductal adenocarcinoma progression.

## 1. Introduction

Pancreatic ductal adenocarcinoma (PDAC) is a devastating disease with the worst survival outcome of any cancer [1]. Its incidence, which is around 10 per 100,000 individuals, is rising in developed countries [2,3], with 458 thousand new cases and 432 thousand deaths in 2018 worldwide [4]. The 5-year survival rate is around 9%, and the 10-year mortality is approaching 99% [5]. Progress towards improving survival has been slow, and current treatment options are inadequate. The only significant progress that has been made is in the form of lower mortality rates for patients eligible for resections, and a slight prolongation and improved quality of life in patients with inoperable disease with the use of chemotherapeutic agents. Single-agent gemcitabine treatment has been the standard of care for inoperable PDAC for many years, although the observed benefits are small in daily practice [6,7,8,9] and seem restricted to patients with a good performance status [10]. More recently, nanoparticle albumin-bound paclitaxel was shown to exert superior antitumor activity compared to gemcitabine monotherapy, thereby establishing nab-paclitaxel and gemcitabine combination therapy as first-line chemotherapy regimens in PDAC [11]. In patients with a good performance status, combination therapy with folinic acid, fluorouracil, irinotecan and oxaliplatin (FOLFIRINOX) is superior over other treatments [12] and FOLFIRINOX is consequently emerging as the new standard of care for relatively fit patients [13]. Importantly however, even in the specific group of patients eligible for FOLFIRINOX treatment, the survival benefit is limited [14].

### 1.1. Tumor Microenvironment of PDAC

PDAC is characterized by a strong desmoplastic reaction, which results in an archetypal tumor microenvironment, consisting of a dense stroma surrounding the tumor cells [15,16]. The stroma forms the bulk of the tumor, taking up to 90% of the total tumor mass and consists of many cellular and acellular components like (myo)fibroblasts, macrophages, blood vessels and extracellular matrix components such as, among others, collagen I, collagen IV, laminin and fibronectin. In the stroma, the extracellular matrix has traditionally been considered to be a stable structure that mainly plays a supportive role in maintaining tissue morphology. Nowadays, however, it is evident that the extracellular matrix forms a dynamic and versatile milieu that affects the fundamental processes of the surrounding cells [17,18]. Accordingly, the loss of extracellular matrix homeostasis and integrity is considered one of the hallmarks of cancer and typically defines transitional events, resulting in cancer progression and metastasis [19]. Moreover, the loss of extracellular matrix homeostasis due to stromal depletion aggravates pancreatic cancer progression in preclinical animal models [20,21,22].

### 1.2. Matrix Metalloproteases in the Tumor Microenvironment

The desmoplastic PDAC stroma contains many different proteases that play a key role in the crosstalk between tumor and stromal cells. An intriguing group of proteases in the tumor microenvironment consist of matrix metalloproteases (MMPs), which are primarily known for their ability to degrade extracellular matrix components. Altered expression and/or activity of MMPs in the tumor microenvironment is likely to lead to the loss of homeostasis of the extracellular matrix, thereby driving PDAC progression. Based upon this notion, MMPs are considered important contributors to PDAC progression and experimental PDAC studies frequently use MMPs as surrogate markers for treatment responses. Decreased MMP levels are, nowadays, considered as important signs of the anti-tumorigenic potential of the gene/compound/miRNA under study. In the current review, we address whether the literature supports the concept that MMPs drive PDAC progression and if decreased MMP levels under experimental settings are indicative of the treatment response. To this end, we performed a systematic review of patient and experimental animal studies, focusing on MMPs in PDAC.

### 1.3. Overview of Matrix Metalloproteases

MMPs are calcium-dependent zinc-containing endopeptidases of the metzincin protease superfamily. They typically contain an N-terminal propeptide of approximately 80–90 amino acids, with a conserved PRCGXPD motif that is responsible for maintaining latency via the binding of the cysteine residue to the zinc atom in the active site [23]. After the proteolytic removal of the propeptide, the active form of MMP contains a calcium-dependent catalytic domain of around 200 amino acids, which contains a hydrophobic S1′-pocket that determines substrate specificity, proceeded by a linker region of variable length, and the C-terminal hemopexin-like domain, which spans approximately 200 amino acids. The hemopexin-like domain, which is absent in some MMP family members, plays a functional role in substrate binding and/or in interactions with tissue inhibitors of metalloproteases (TIMPs), a family of specific MMP protein inhibitors [24].

Since the identification of a diffusible collagenolytic factor in living amphibian tissue that is capable of degrading undenatured calf skin collagen [25], a total of 24 MMPs have been identified in humans [26]. According to their substrate specificity, MMPs are classified into subfamilies: (1) collagenases, (2) gelatinases, (3) stromelysins, (4) matrilysins, (5) membrane-type MMPs and (6) others. Despite the general acceptance of the classification system based on extracellular matrix substrates, MMPs are rather promiscuous in substrate recognition and also proteolytically cleave substrates beyond extracellular matrix proteins.

## 2. Methods

To provide a comprehensive overview of the role of MMPs in PDAC, a systematic PubMed search without restrictions was performed. A combination of the search terms “pancreatic cancer” and every individual MMP (both using the official gene name and the common name; see Supplementary Materials Table S1) was used to retrieve papers published up to 1 March 2020. All papers were independently screened by their title and abstract, followed by full text assessment to include papers that contained MMP expression analysis in PDAC patients and papers that contained animal experiments that targeted (either genetically or pharmacologically) MMPs in pancreatic cancer models. The excluded papers were those that contained in vitro data only, papers that assayed MMP levels in experimental animal models without interventions or genetic modifications, or papers that did not focus on PDAC.

## 3. Results

**Figure 1:**
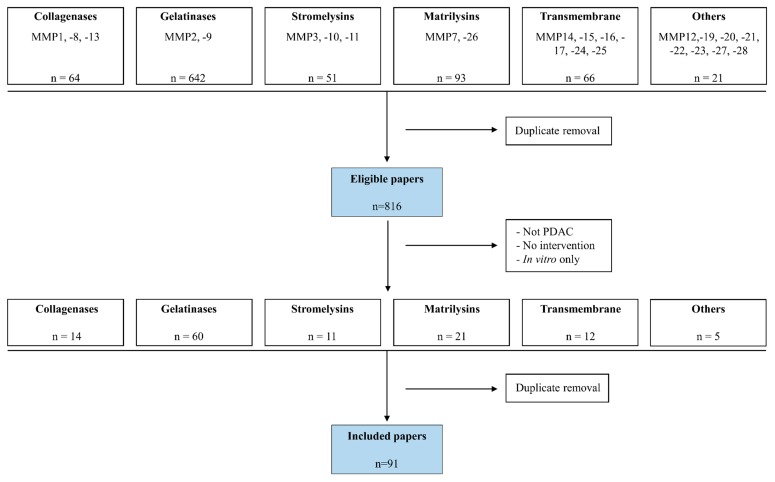
Flowchart of paper inclusion. Using the search criteria indicated in Supplementary Materials Table S1, we obtained 814 eligible papers that we screened for the presence of patient and/or matrix metalloprotease (MMP) intervention in animal models. After the exclusion of duplicate papers, we ended up with 91 papers that were included in the review.

We retrieved 64 papers focusing on collagenases, 642 papers focusing on gelatinases, 51 papers focusing on stromelysins, 93 papers focusing on matrilysins, 66 papers focusing on transmembrane MMPs and 21 papers focusing on other MMPs (Figure 1). After the removal of duplicates, 816 eligible studies were identified and were vigorously screened to obtain those that contained patient data and/or animal experiments in which MMPs were targeted. This resulted in the inclusion of 14 papers focusing on collagenases, 60 on gelatinases, 11 on stromelysins, 21 on matrilysins, 12 on transmembrane-type MMPs and five on the so-called “other” MMPs. As several of the eligible papers contained data on multiple MMPs, the total number of papers including patient/experimental animal data selected for the review was 91.

### 3.1. Collagenases in PDAC

Despite the general notion that collagenases (MMP1, MMP8 and MMP13) are key players in cancer biology [27,28,29], relatively little is known about collagenases in PDAC. Although MMP-1 is consistently shown to be overexpressed in PDAC patients compared to healthy controls [30,31,32,33,34,35,36], its effects on cancer progression are inconsistent (Table 1). For example, MMP1 overexpression has been reported as being associated with both a poor prognosis [30] and prolonged survival [37], although no correlations with tumor size, differentiation status and lymph node involvement have been observed [30,36,38]. Despite an elegant recent study showing that MMP1-dependent protease activated receptor (PAR)-1 drives PDAC cell migration and perineural invasion [33], the important role of MMP1 in PDAC is not supported by the experimental data. Besides MMP1 overexpression, MMP8 [36,39] and MMP13 [34,40] are also overexpressed in PDAC patients compared to healthy controls. The relevance of increased MMP expression is not well documented and only a single study showed that MMP-13 expression is associated with lymph node metastasis and the tumor’s pathological stage [41]. Interestingly however, MMP13 overexpression significantly promoted the invasion of the PDAC cells in vitro, whereas MMP13 inhibition blocked leptin-mediated PDAC cell invasion [41], while CD40 agonist-dependent resolution of fibrosis and enhanced chemotherapy efficacy were diminished by MMP13 inhibition [42].

### 3.2. Gelatinases in PDAC

The most studied MMPs in PDAC are, without a doubt, the gelatinases (MMP2 and MMP9; see Figure 1). The vast majority of studies show that both MMP2 [34,35,43,44,45,46,47,48,49,50,51,52,53,54,55,56,57,58,59,60,61,62] and MMP9 [34,36,39,48,49,53,54,59,62,63,64,65,66,67] are upregulated in PDAC patients (Table 1), while a minority of studies fail to show a difference in expression between PDAC and the controls [36,38,52,60,61,68,69,70,71]. The potential clinical relevance is less pronounced, as just half of the studies reported associations between increased MMP2 or MMP9 levels with clinical characteristics such as survival, metastasis or tumor stage [43,46,47,48,50,51,53,56,57,58,61,63,65,67,68,72,73], whereas in the other half of the studies no such correlations were observed (Table 2). Despite the rather diverse observations in patients, initial preclinical experimental animal experiments showed promising results (Table 3). Batimastat treatment of mice harboring orthotopic pancreatic cancers reduced cancer growth, metastasis and death compared to control-treated mice, while also potentiating gemcitabine sensitivity [74,75,76,77]. Batimastat was also shown to reduce metastasis and death when PDAC cells were directly injected into the spleen of recipient mice, in order to mimic liver metastasis in PDAC [78]. Although batimastat is not specific to MMP2 and MMP9 and also inhibits MMP1, MMP3, MMP7, MMP8 and several ADAM family members, based on the gelatin zymography of tumor samples before and after treatment, it was hypothesized that the tumor-inhibiting effect of batimastat was dependent on MMP2 and, to a lesser extent, MMP9. The potential importance of MMP2 and MMP9 in PDAC progression is further supported by studies using more specific inhibitors like MMI-166, RO28-2653 and OPB-3206. Indeed, the selective MMP2, MMP9 and MMP14 inhibitor MMI-166 inhibited PDAC growth in both mice and Syrian hamsters [79,80], whereas RO28-2653 and OPB-3206 (both also selective MMP2, MMP9 and MMP14 inhibitors) reduced chemically induced pancreatic carcinogenesis in Syrian hamsters [81,82]. Finally, treatment with the selective MMP2 and MMP9 inhibitor SB-3CT reduced the lung metastasis of subcutaneously implanted PDAC cells [83].

The most conclusive evidence of the role of MMP2 in PDAC progression comes from subcutaneous models, in which the injection of shMMP2-silenced PANC1 cells resulted in smaller tumors compared to the injection of control shRNA transduced cells [84], whereas treatment with MMP2-blocking peptides limited tumor growth and angiogenesis [85].

In a similar way to the inconclusive association studies in patients (see above and Table 2), experimental animal experiments specifically targeting MMP9 show inconsistent results (Table 3). Orthotopic injections of MMP9-overexpressing Panc02 cells led to bigger tumors than injections of their control counterparts, but the absence/presence of MMP9 did not affect metastasis [86]. Treatment with a MMP9-blocking antibody did not affect the tumor growth of subcutaneously implanted PDAC cells, but did enhance gemcitabine and nab-paclitaxel sensitivity when PDAC cells were injected into the peritoneal cavity [87]. Doxycycline treatment, suggested to specifically target MMP9, reduced the growth of subcutaneously injected Capan-1 cells [88]. Finally, subcutaneous or orthotopic implantation of PDAC cells in MMP9-deficient mice diminished tumor take, tumor growth, angiogenesis and metastasis [83,89] but tumor progression and metastasis increased in MMP9-deficient mice on the *Kras(G12D)*/*Tp53* background [90].

### 3.3. Stromelysins in PDAC

Clinical studies do not support the general role of stromelysins (MMP3, MMP10 and MMP11) in PDAC (Table 1). Although MMP11 is consistently upregulated and associated with clinical characteristics in PDAC patients [35,36,91,92,93], the data for MMP3 is more controversial. Only half of the studies focusing on MMP3 suggest its expression is increased in PDAC patients compared to control tissue [34,35,94,95], and only a single study suggests that MMP3 is associated with patient survival [95]. Besides clinical studies, preclinical animal models also do not support an important role for stromelysins in PDAC progression. Apart from a study which suggests, but does not prove, that MMP10 drives the invasion and metastasis of PDAC [96], it has only been shown that MMP3 overexpression on the *Kras(G12D)* background increases neoplastic alterations in pancreatic acinar cells [94]. These premalignant morphological changes were accompanied by the recruitment of infiltrating immune cells and the expression of smooth muscle actin and collagen, indicating that MMP3 is not only a coconspirator of Kras in inducing tumorigenic changes in epithelial cells, but also that it promotes the establishment of a tumorigenic microenvironment. Though it has been suggested that MMP3 may play a role in PDAC initiation, the actual importance of endogenous MMP3 (as opposed to overexpressed MMP3) in PDAC progression and its potential clinical relevance remains elusive.

### 3.4. Matrilysins in PDAC

MMP7 and MMP26 are the only two members of the matrilysin subfamily. A large number of studies have compared MMP7 expression in PDAC patients with pancreatitis patients and/or healthy controls and have consistently shown that MMP7 levels are elevated in PDAC patients (Table 1) [34,35,36,54,69,91,97,98,99,100,101,102,103,104]. More importantly, MMP7 levels correlate with metastasis and/or survival in most, but not all, studies. Based upon these reports, it is suggested that MMP7 is an important regulator of tumor formation. In line with this notion, preclinical experimental animal models show that MMP7 expression is intimately linked with acinar-to-ductal metaplasia and that pancreatic duct ligation-dependent acinar cell loss, caspase-3 activation, and subsequent metaplasia is significantly reduced in MMP7-deficient mice (Table 3) [98]. The effect of MMP7 on acinar-to-ductal metaplasia seems model-specific, however, as MMP7 deficiency did not affect pancreatitis driven-PanIN development in Pfta1-Cre Kras(G12D) mice [105]. In addition to PDAC initiation, MMP7 also seems to drive PDAC progression. Using several genetic Kras-driven PDAC models, it was shown that both tumor size and metastasis were significantly reduced by MMP7 deficiency. The percentage of mice with lymph node metastasis reduced from around 60 in MMP7-proficient mice to 0 in MMP7-deficient mice, whereas the percentage of mice with liver metastasis dropped from 67% to 13% due to MMP7 deficiency [105]. In line with these findings, the metastasis of MMP7-silenced PANC1 cells was largely reduced compared to control PANC1 cells, whereas pharmacological MMP7 inhibition with sulfur-2-(4-chlorine-3-trifluoromethyl phenyl)-sulfonamido-4-phenylbutyric acid (SCTPSPA) also significantly reduced the metastasis of PANC1 cells [101]. MMP26 expression was also induced in PDAC patients compared to the controls and, intriguingly, MMP26 was expressed significantly more often in tumors with lymph node involvement. Although this is suggestive of the general role of matrilysins in PDAC progression, experimental data confirming the pro-tumorigenic role of MMP26 in PDAC is lacking and it remains to be established whether MMP26 is indeed a driver of disease progression or merely acts as a marker of PDAC metastasis [106].

### 3.5. Membrane-Type MMPs in PDAC

Seven membrane-bound MMPs have been described so far: the transmembrane members MMP14, MMP15, MMP16, MMP23 and MMP24, and the GPI-anchored members MMP17 and MMP25. Of the membrane-bound MMPs, MMP14 seems most relevant in the setting of PDAC (Table 1, Table 2 and Table 3). Indeed, the overexpression of MMP14 in mice expressing an activating Kras(G12D) mutation led to more large, dysplastic mucin-containing papillary lesions compared to the control Kras(G12D) mice (Table 3) [107]. Using subcutaneous models, MMP14 overexpression in cancer cells seems to reduce the cytotoxic effect of gemcitabine [108], whereas MMP14 inhibition in pancreatic stellate cells limits tumor growth [84]. Moreover, the cancer cell-specific overexpression of membrane-type 1 matrix metalloproteinase cytoplasmic tail binding protein-1 (MTCBP-1; MMP14 binding protein inhibiting its activity) restricts metastasis in orthotopic PDAC models, further suggesting that MMP14 may enhance tumor progression [109]. However, clinical data do not support the important role of MMP14 in PDAC progression (Table 1 and Table 2). Although MMP14 may be overexpressed in PDAC [44,110], MMP14 does not correlate with clinical characteristics such as tumor differentiation, tumor size, lymph node status, or patient survival [31,37,111].

### 3.6. Other MMPs in PDAC

The so-called other MMPs (i.e., MMP12, MMP19, MMP20, MMP21, MMP27 and MMP28) are not very well characterized in PDAC. Although some members seem to be overexpressed in PDAC [106,111,112] and may be associated with tumor stage and patients survival (Table 1) [111,112,113], no preclinical studies have addressed the role of these MMPs in PDAC (Table 2). Therefore, their actual importance remains to be established.

### 3.7. Clinical Trials with MMP Inhibitors in PDAC

Only two phase 3 trials focusing on MMP inhibition in PDAC have been published [114,115]. One trial showed that the addition of marimastat (a broad-spectrum MMP inhibitor targeting MMP1, MMP2, MMP7, MMP9 and MMP14) to gemcitabine in a double-blind placebo-controlled, randomized study was well-tolerated but did not show clinical benefits in PDAC patients [114]. The overall response rates (11% and 16% with and without the addition of marimastat, respectively), progression-free survival and time to treatment failure were similar in both treatment arms. Another phase 3 trial showed that BAY 12-9566 (tanomastat; MMP2, MMP3 and MMP9 inhibitor) treatment was also well tolerated by PDAC patients but was inferior to gemcitabine, with median survival times of 3.74 and 6.59 months for the BAY 12-9566 and gemcitabine arm, respectively [115]. Median progression-free survival and quality-of-life analyses also favored gemcitabine, arguing against MMP inhibition in the setting of PDAC.

The fact that there are no clinical benefits obtained through MMP inhibition does not imply that MMPs do not contribute to PDAC progression. As elegantly discussed [116,117], the disappointing clinical trial results may be due to several reasons, of which the inclusion of advanced stage disease seems most relevant. Broad spectrum MMP inhibitors may also lack efficacy as they could block the potential tumor inhibitory activities of specific MMPs. As indicated above, MMP9 deficiency on the *Kras(G12D)* background enhanced tumor progression and invasive growth [90], supporting this notion and providing an alternative explanation for the negative marimastat and BAY 12-9566 results in PDAC patients. Finally, the poor clinical efficacy of MMP inhibitors could also be explained by the overestimation of the role of MMPs in PDAC progression based on preclinical models that do not fully capture the complexity of human disease.

## 4. Conclusions

The potential clinical relevance of MMPs in PDAC has largely been addressed using patient-derived tumor material. These studies show a rather consistent picture with respect to MMP overexpression in tumors compared to control sections, although almost 25% of the studies do not show significant differences between patients and controls. However, the association of MMP overexpression with clinical characteristics is not as convincing as suggested in the literature. Half of the studies show that high MMP levels are associated with (lymph node) metastasis and reduced survival, whereas the other half of the studies do not show any correlation with clinical characteristics. Patient-derived data do not, therefore, seem to allow firm conclusions that MMP expression levels (in general) are associated with PDAC progression and poor prognosis to be drawn, especially when considering that publication bias may have resulted in negative studies not being published.

Initial preclinical experimental animal models using broad spectrum MMP inhibitors are more in line with the general role of MMPs in PDAC progression, as different inhibitors limit tumor growth and metastasis in subcutaneous, orthotopic and spontaneous PDAC models. The contribution of individual MMPs in PDAC progression is, however, not very well established. Only MMP2, MMP7 and MMP14 are shown to potentiate tumor growth and/or metastasis in multiple independent papers. For others, the literature is conflicting or missing and no clear conclusions can be drawn. Importantly, however, conflicting results do not indicate that the individual MMPs have no effect in PDAC. The biology of PDAC and MMP is complex and MMPs may act in a context-dependent manner, with both tumor-promoting and tumor-inhibiting effects. The conflicting role of MMP9 serves as an excellent example for this notion. The data rather convincingly show that tumor MMP9 expression drives PDAC progression, but systemic MMP9 ablation triggers invasive growth and metastasis by blocking MMP9-dependent tumor-inhibiting effects in the bone marrow.

Despite the presence of a large range of MMP-deficient animals and the relative ease of generating MMP deficient cells with CRISPR technology, the majority of MMPs have not been studied in preclinical PDAC animal models. To fully appreciate the importance of individual MMPs in PDAC progression and to assess their potential clinical relevance, we have to await studies that combine (pharmacological inhibition in) genetic Kras-driven spontaneous models with subcutaneous and/or orthotopic models, in which MMPs are specifically depleted in stromal or tumor cells. In particular, experiments that address pharmacological treatment with specific MMP inhibitors after tumors could turn out to be invaluable for establishing the context-dependent role of individual MMPs in PDAC. Before such studies have been performed, we should be careful not to generalize the available literature.

Although broad spectrum MMP inhibitors limit PDAC progression in preclinical animal models [73,74,75,76,77,78,79,80,81,82], they seem to lack efficacy in a clinical setting [115,116]. This disparity between preclinical data and clinical trials can be attributed to several factors—for instance, differences in pharmacokinetics, pharmacodynamics and metabolism and the failure to accurately model the tumor microenvironment [128]. In particular, xenograft models, which lack a functional immune system, show a reduced complexity and cellular diversity compared to human disease models. Moreover, the degree of aneuploidy in human tumors results in great variety within inter-tumoral gene modifications, in a different manner compared to how it occurs in mice [129,130]. All of these species-related differences limit the capacity of preclinical mouse models to accurately predict the response of MMP inhibitors in PDAC patients.

In conclusion, based on our systematic review on the role of matrix metalloproteases in PDAC, we conclude that the available literature is not as consistent as envisioned and that, although individual matrix metalloproteases seem to contribute to PDAC growth and metastasis, our review does not support the generalized notion that matrix metalloproteases drive PDAC progression.

## Figures and Tables

**Table 1:** MMP expression levels in Pancreatic ductal adenocarcinoma (PDAC) patients and controls. Red indicates increased MMP levels, blue indicates no difference and green indicates decreased MMP levels in PDAC patients.

Member	Patient Number	Method	Difference	Reference
MMP1	45 PC, 10 CO	IHC	no difference	[36]
MMP1	8 PC, 8 CO	RNA	no difference	[59]
MMP1	248 PC, 216 CO	Serum	no difference	[69]
MMP1	46 PC, 5 CO	IHC	up vs healthy	[30]
MMP1	25 PC	RNA	up vs adjacent CO	[31]
MMP1	10 PC, 12 CP, 5 CO	IHC	up vs CO	[32]
MMP1	45 PC	RNA	up vs adjacent CO	[33]
MMP1	30 PC	IHC	up vs adjacent CO	[33]
MMP1	104 PC, 62 CO	IHC	up vs CO	[34]
MMP1	17 PC, 17 CO	RNA	up vs CO	[35]
MMP1	18 PC, 8 CO	RNA	up vs healthy	[36]
MMP2	75 PC, 10 CO	IHC	no difference	[36]
MMP2	18 PC, 8 CO	RNA	no difference	[36]
MMP2	70 PC and 10 CO	IHC	no difference	[38]
MMP2	92 PC, 43 CP, 91 CO	Serum	no difference	[68]
MMP2	35 PC	RNA/IHC	no difference	[70]
MMP2	46 PC, 13 CO	Serum	no difference	[71]
MMP2	104 PC, 62 CO	IHC	up vs CO	[34]
MMP2	17 PC, 17 CO	RNA	upvsCO	[35]
MMP2	122 PC	IHC	up vs adjacent CO	[43]
MMP2	18 PC, 9 CP, 9 CO	RNA	up vs both others	[44]
MMP2	12 PC, 11 CP, 7 CO	pancreatic juice	up vs both others	[45]
MMP2	127 PC	IHC	up vs CO	[46]
MMP2	20 PC	IHC	up vs CO	[47]
MMP2	32 PC, 31 CP	ELISA on tissue	up vs CP	[48]
MMP2	110 PC, 24 BT	Plasma	up vs BT	[49]
MMP2	37 PC, 7 CP	IHC	up vs CP and CO	[50]
MMP2	45 PC	IHC	up vs CO	[51]
MMP2	51 PC, 60 CO	Urine	up vs CO	[52]
MMP2	44 PC, 8 CO	IHC	up vs CO	[52]
MMP2	30 PC, 17 CO	IHC	up vs CO	[53]
MMP2	29 PC	IHC	up vs adjacent CO	[54]
MMP2	127 PC, 25 CP, 25 CO	Plasma	up vs CP and CO	[55]
MMP2	106 PC	RNA/WB	up vs adjacent CO	[56]
MMP2	40 PC, 10 CO	IHC	up vs CO	[57]
MMP2	67 PC, 20 CO	IHC	up vs adjacent CO	[58]
MMP2	8 PC, 8 CO	RNA	upvsCO	[59]
MMP2	10 PC, 3 CO	ZG	upvsCO	[60]
MMP2	33 PC, 14 CP, 13 CO	ZG/WB	upvsCO	[61]
MMP2	22 PC, 9 CP, 9 CO	RNA	up vs adjacent CO	[62]
MMP2	10 PC, 213 CO	Serum	down vs CO	[118]
MMP3	45 PC, 10 CO	IHC	no difference	[36]
MMP3	18 PC, 8 CO	RNA	no difference	[36]
MMP3	8 PC, 8 CO	RNA	no difference	[59]
MMP3	104 PC, 62 CO	IHC	up vs CO	[34]
MMP3	17 PC, 17 CO	RNA	up vs CO	[35]
MMP3	140 PC, 12 CO	IHC	up vs CO	[94]
MMP3	140 PC, 12 CO	IHC	up vs CO	[95]
MMP7	18 PC, 8 CO	RNA	no difference	[36]
MMP7	104 PC, 62 CO	IHC	up vs CO	[34]
MMP7	17 PC, 17 CO	RNA	up vs CO	[35]
MMP7	45 PC, 10 CO	IHC	up vs CO	[36]
MMP7	29 PC	IHC	up vs adjacent CO	[54]
MMP7	248 PC, 216 CO	Serum	up vs CO	[68]
MMP7	44 PC, 17 CP	RNA	up vs CP	[91]
MMP7	70 PC	RNA	up vs adjacent CO	[97]
MMP7	32 PC, ? CO	IHC	up vs CO	[98]
MMP7	47 PC, 10 CO	IHC	up vs CO	[99]
MMP7	63 PC, 31 CP	Plasma	up vs CP	[100]
MMP7	30 PC	RNA	up vs adjacent CO	[101]
MMP7	5 PC, 5 CP, 62 CO	IHC	up vs CP and CO	[102]
MMP7	131 PC, 30 CP, 131 CO	Plasma	up vs CO	[103]
MMP7	10 PC	RNA	up vs adjacent CO	[104]
MMP8	248 PC, 216 CO	Serum	no difference	[69]
MMP8	75 PC, 10 CO	IHC	up vs CO	[36]
MMP8	91 PC, 41 CP, 30 CO	RNA (PBMCs)	up vs CO	[39]
MMP9	18 PC, 8 CO	RNA	no difference	[36]
MMP9	70 PC, 10 CO	IHC	no difference	[38]
MMP9	51 PC, 60 CO	urine	no difference	[52]
MMP9	10 PC, 3 CO	ZG	no difference	[60]
MMP9	33 PC, 14 CP, 13 CO	ZG/WB	no difference	[61]
MMP9	248 PC, 216 CO	Serum	no difference	[69]
MMP9	35 PC	RNA/IHC	no difference	[70]
MMP9	104 PC, 62 CO	IHC	up vs CO	[34]
MMP9	45 PC, 10 CO	IHC	up vs CO	[36]
MMP9	91 PC, 41 CP, 30 CO	RNA (PBMCs)	up vs CP and CO	[39]
MMP9	32 PC, 31 CP	ELISA on tissue	up vs CP	[48]
MMP9	110 PC, 24 BT	Plasma	up vs BT	[49]
MMP9	30 PC, 17 CO	IHC	up vs CO	[53]
MMP9	29 PC	IHC	up vs adjacent CO	[54]
MMP9	8 PC, 8 CO	RNA	up vs CO	[59]
MMP9	22 PC, 9 CP, 9 CO	RNA	up vs adjacent CO	[62]
MMP9	36 PC	IHC	up vs CO	[63]
MMP9	9 PC, 9 CO	MS/MS	up vs CO	[64]
MMP9	78 PC, 45 CP, 70 CO	Serum	up vs both	[65]
MMP9	62 PC, 16 CO	IHC	up vs CO	[66]
MMP9	103 PC, 6 CO	IHC	up vs CO	[67]
MMP10	17 PC, 17 CO	RNA	no difference	[35]
MMP11	17 PC, 17 CO	RNA	up vs CO	[35]
MMP11	18 PC, 8 CO	RNA	up vs CO	[36]
MMP11	75 PC, 10 CO	IHC	up vs CO	[36]
MMP11	44 PC, 17 CP	RNA	up vs CP	[91]
MMP11	12 PC, 16 CO	Blood	up vs CO	[92]
MMP11	21 PC, 9 CO	IHC	up vs CO	[93]
MMP12	75 PC, 10 CO	IHC	no difference	[36]
MMP12	39 PC, 13 CO	RNA/WB/IHC	up vs CO	[111]
MMP13	104 PC, 62 CO	IHC	up vs CO	[34]
MMP13	45 PC	RNA	up vs adjacent CO	[40]
MMP14	75 PC, 10 CO	IHC	no difference	[36]
MMP14	35 PC	RNA/IHC	no difference	[111]
MMP14	18 PC, 9 CP, 9 CO	RNA	up vs both others	[44]
MMP14	64 PC, 9 CO	IHC	up vs CO	[110]
MMP15	18 PC, 9 CP, 9 CO	RNA	up vs both others	[44]
MMP15	18 PC, 8 CO	RNA	reduced vs CO	[36]
MMP16	18 PC, 9 CP, 9 CO	RNA	no difference	[44]
MMP16	12 PC	IHC	up vs adjacent CO	[119]
MMP19	102 PC	IHC	up vs adjacent CO	[112]
MMP20	102 PC	IHC	up vs adjacent CO	[112]
MMP21	25 PC, 18 CO	IHC	up vs CO	[106]
MMP26	25 PC, 18 CO	IHC	up vs CO	[106]

Pancreatic cancer (PC); pancreatitis (CP); healthy control (CO); benign tumor (BT); immunohistochemistry (IHC); Western blot (WB); zymography (DG).

**Table 2:** Association between MMP expression and clinical characteristics of PDAC. Red indicates that MMP levels are associated with poor outcome, blue indicates no association and green indicates that MMP levels are associated with improved survival.

Member	Patient Number	Method	Correlation		Reference
MMP1	45 PC, 10 CO	IHC	no		[36]
MMP1	70 PC	IHC	no		[38]
MMP1	46 PC, 5 CO	IHC	OS,	LM, Size, Stage	[30]
MMP1	30 PC	IHC	PNI		[33]
MMP1	51 PC	IHC/serum	OS		[37]
MMP2	75 PC, 10 CO	IHC	no		[36]
MMP2	70 PC, 10 CO	IHC	no		[38]
MMP2	51 PC	IHC/serum	no		[37]
MMP2	32 PC, 31 CP	ELISA on tissue	no		[48]
MMP2	37 PC, 7 CP	IHC	no		[50]
MMP2	29 PC	IHC	no		[54]
MMP2	127 PC, 25 CP, 25 CO	plasma	no		[55]
MMP2	35 PC	RNA/IHC	no		[70]
MMP2	32 PC	IHC	no		[120]
MMP2	67 PC	IHC	LM,	PNI, OS, DF	[121]
MMP2	122 PC	IHC	OS, DF		[43]
MMP2	127 PC	IHC	OS, Stage		[46]
MMP2	20 PC	IHC	LM		[47]
MMP2	37 PC, 7 CP	IHC	LM, DM		[50]
MMP2	45 PC	IHC	OS, LM, Stage		[51]
MMP2	30 PC, 17 CO	IHC	LM, Stage, Size		[53]
MMP2	106 PC	RNA/WB	DM, Stage		[56]
MMP2	40 PC, 10 CO	IHC	LM		[57]
MMP2	67 PC, 20 CO	IHC	LM, Stage, PNI		[58]
MMP2	33 PC, 14 CP, 13 CO	ZG/WB	Stage		[61]
MMP2	92 PC, 43 CP, 91 CO	serum	LM, DM		[68]
MMP2	32 PC	IHC	VI		[72]
MMP2	88 PC	IHC	OS		[73]
MMP3	45 PC, 10 CO	IHC	no		[36]
MMP3	18 PC, 8 CO	RNA	no		[36]
MMP3	70 PC	IHC	no		[38]
MMP3	140 PC, 12 CO	IHC	OS		[95]
MMP7	51 PC	IHC/serum	no		[37]
MMP7	29 PC	IHC	no		[54]
MMP7	88 PC	IHC	no		[73]
MMP7	45 PC, 10 CO	IHC	OS,	LM, DIF, Stage	[36]
MMP7	70 PC	IHC	OS,	Size, DIF	[38]
MMP7	134 PC	IHC	Stage, PNI,	OS	[122]
MMP7	70 PC	RNA	LM, Size		[97]
MMP7	47 PC, 10 CO	IHC	OS, DM		[99]
MMP7	10 PC	RNA	OS		[104]
MMP7	101 PC	serum	OS		[105]
MMP7	39 PC	IHC	LM, OS		[123]
MMP8	75 PC, 10 CO	IHC	no		[36]
MMP8	91 PC, 41 CP, 30 CO	RNA (PBMCs)	no		[39]
MMP9	45 PC, 10 CO	IHC	no		[36]
MMP9	70 PC, 10 CO	IHC	no		[38]
MMP9	51 PC	IHC/serum	no		[37]
MMP9	91 PC, 41 CP, 30 CO	RNA (PBMCs)	no		[39]
MMP9	29 PC	IHC	no		[54]
MMP9	33 PC, 14 CP, 13 CO	ZG/WB	no		[61]
MMP9	9 PC, 9 CO	MS/MS	no		[64]
MMP9	35 PC	RNA/IHC	no		[70]
MMP9	32 PC	IHC	no		[123]
MMP9	27 PC	IHC	no		[124]
MMP9	62 PC, 16 CO	IHC	PNI,	LM, Stage, Size	[66]
MMP9	63 PC	IHC	VI,	OS, LM, DM	[125]
MMP9	62 PC	IHC	LM,	OS	[126]
MMP9	32 PC, 31 CP	ELISA on tissue	LM		[48]
MMP9	30 PC, 17 CO	IHC	LM, Stage, Size		[53]
MMP9	36 PC	IHC	LM, DM		[63]
MMP9	78 PC, 45 CP, 70 CO	serum	OS		[65]
MMP9	103 PC, 6 CO	IHC	OS, LM, DM, VI, Stage		[67]
MMP9	32 PC	IHC	VI		[72]
MMP9	88 PC	IHC	OS, DF,	DM	[73]
MMP10	51 PC	IHC/serum	no		[37]
MMP11	75 PC, 10 CO	IHC	OS, LM,	DIF, Size	[36]
MMP11	not indicated	RNA	OS		[92]
MMP12	75 PC, 10 CO	IHC	no		[36]
MMP12	39 PC, 13 CO	RNA/WB/IHC	OS,	LM, Stage	[111]
MMP13	60 PC	IHC	LM		[41]
MMP14	70 PC	IHC	no		[38]
MMP14	75 PC, 10 CO	IHC	no		[36]
MMP14	37 PC	RNA/IHC	no		[70]
MMP15	78 PC	IHC	OS, DF,	PNI, LM, DM, Stage	[127]
MMP19	102 PC	IHC	OS, DF, PNI, Stage		[112]
MMP20	102 PC	IHC	OS, DF, Stage, PNI		[112]
MMP21	25 PC, 18 CO	IHC	no		[106]
MMP26	25 PC, 18 CO	IHC	LM		[106]
MMP28	not indicated	RNA	OS		[113]

Pancreatic cancer (PC); pancreatitis (CP); healthy control (CO); benign tumor (BT); immunohistochemistry (IHC); Western blot (WB); zymography (DG); overall survival (OS); disease-free survival (DF); lymph node metastasis (LM); perineural invasion (PNI); venous invasion (VI); distant metastasis (DM); differentiation (DIF).

**Table 3:** Experimental animal models that target MMPs.

Target	Model	“Treatment”	Result	Reference
**MMP1**	Sciatic nerve invasion	shMMP1 PANC1 cells	Reduced perineural invasion	[33]
**MMP2/9?**	Orthotopic injection HPAC cells	Batimastat (day −7 till death/sacrifice)	Increased gemcitabine sensitivity, No effect single treatment	[74]
	Orthotopic injection HPAC cells	Batimastat (day −4 till death/sacrifice)	Reduced tumor growth, metastasis and death	[75]
	Orthotopic injection HPAC cells	Batimastat (day 7 till death/sacrifice)	Reduced local invasion and death	[76]
	Orthotopic injection HPAC cells	Batimastat (day 7 till death/sacrifice)	Reduced tumor weight	[77]
	Injection AsPC1 or Capan-1 cells in spleen	Batimastat (day −7 till day 14)	Reduced metastasis and death	[78]
	Subcutaneous injection SW1990 cells	MMI-166 from day 7 till sacrifice at day 28	Reduced tumor growth	[79]
	Orthotopic injection PGHAM cells (Syrian hamster)	MMI-166 (day 1 till sacrifice)	Reduced tumor growth, liver metastasis and MVD	[80]
	BOP injections (Syrian hamster)	RO28-2653 (week 6 till week 14)	Reduced liver metastasis, No effect death	[81]
	BOP injections (Syrian hamster)	OPB-3206 in diet from day 48 till sacrifice	Reduced invasive carcinoma	[82]
	Subcutaneous injection Panc02 or MIAPaca2 cells	SB-3CT (day 1 till sacrifice)	Reduced lung metastasis	[83]
**MMP2**	Subcutaneous injection organoid and PSC	shMMP2 PSC	Reduced tumor growth	[84]
	Subcutaneous injection PANC-1 or CFPAC-1 cells	MMP2 blocking peptides after tumor take	Reduced growth and MVD	[85]
**MMP3**	Kras(G12D) mice	MMP3 overexpression	Increased neoplastic alterations	[94]
**MMP7**	Ductal ligation	MMP7 deficient mice	Reduced ductal metaplasia	[98]
	Pfta1-Cre/KrasG12D mice	MMP7 deficiency	No effect acinar to ductal metaplasia	[105]
	Pdx1-Crelate/KrasG12D mice	MMP7 deficiency	Reduced tumor development	[105]
	Pdx1-CreLate/KrasG12D/p53f/+ mice	MMP7 deficiency	Reduced tumor growth and metastasis	[105]
	Tail vein injection PANC1 cells	shMMP7	Reduced liver and lung metastasis	[101]
		SCTPSPA (day −2 till day 25)	Reduced lung metastasis	[101]
**MMP9**	Subcutaneous injection Panc02 cells	MMP9 deficient mice	Reduced lung metastasis	[83]
	Orthotopic injection Panc02 cells	MMP9 overexpression	Enhanced tumor growth, No effect metastasis	[86]
	Subcutaneous injection AsPC-1 cells	aMMP9 antibody AB0046 (day 1 till day 14)	No effect on tumor weight	[87]
	Injection AsPC-1 cells in peritoneal cavity	aMMP9 antibody AB0046 (day 14 till day 56)	Increased gemcitabine/nab-paclitaxel sensitivity, No effect metastasis	[87]
	Subcutaneous injection Capan-1 cells	Doxycycline (day 1 till day 14)	Reduced growth and MVD	[88]
	Orthotopic injection L3.6pl cells	MMP9 deficient mice	Reduced tumor take, growth and MVD	[89]
	Pdx-1+/Cre;KrasG12D;Trp53 mice	MMP9 deficiency	Increased progression and invasive growth	[90]
	Intravenous injection 9801 or Panc02 cells	MMP9 deficient mice	Increased metastasis	[90]
**MMP14**	Subcutaneous injection organoid and PSC	shMMP14 PSC	Reduced tumor growth	[84]
	KrasG12D mice	MMP14 overexpression	Increased number of PanIN lesions	[107]
	Subcutaneous injection PANC1 or HPAF-II cells	MMP14 overexpression	Reduced gemcitabine sensitivity, No effect single treatment	[108]
	Orthotopic injection DanG or BxPc3 cells	MTCBP-1 overexpression	Reduced metastasis, No effect tumor growth	[109]

Note: All experiments were performed using mice unless indicated otherwise.

## Supplementary Materials

The following are available online at https://www.mdpi.com/2079-7737/9/4/80/s1, Table S1: Search terms used and number of papers retrieved.
