# Supplementary material for: Matrix Metalloproteases in Pancreatic Ductal Adenocarcinoma: Key Drivers of Disease Progression?
Source: Biology (Basel). 2020 Apr 18;9(4):80. doi: 10.3390/biology9040080 (PMC7235986; doi:10.3390/biology9040080)
Supplement: Supplementary file 1 [file biology-09-00080-s001.pdf]

Supplemental Table 1: Search terms used and number of papers retrieved.

Family	Query in Pubmed	# of papers
Collagenases	Pancreatic cancer AND MMP1	45
	Pancreatic cancer AND collagenase 1	1
	Pancreatic cancer AND MMP8	5
	Pancreatic cancer AND collagenase 2	6
	Pancreatic cancer AND MMP13	14
	Pancreatic cancer AND collagenase 3	9
Gelatinases	Pancreatic cancer AND MMP2	357
	Pancreatic cancer AND gelatinase A	269
	Pancreatic cancer AND MMP9	397
	Pancreatic cancer AND gelatinase B	200
Stromelysins	Pancreatic cancer AND MMP3	28
	Pancreatic cancer AND stromelysin-1	20
	Pancreatic cancer AND MMP10	7
	Pancreatic cancer AND stromelysin-2	4
	Pancreatic cancer AND MMP11	8
	Pancreatic cancer AND stromelysin-3	7
Matrilysins	Pancreatic cancer AND MMP7	81
	Pancreatic cancer AND matrilysin	59
	Pancreatic cancer AND MMP26	2
	Pancreatic cancer AND matrilysin-2	0
Transmembrane MMPs	Pancreatic cancer AND MMP14	29
	Pancreatic cancer AND MT1-MMP	49
	Pancreatic cancer AND MMP15	2
	Pancreatic cancer AND MT2-MMP	6
	Pancreatic cancer AND MMP16	6
	Pancreatic cancer AND MT3-MMP	5
	Pancreatic cancer AND MMP17	0
	Pancreatic cancer AND MT4-MMP	0
	Pancreatic cancer AND MMP24	1
	Pancreatic cancer AND MT5-MMP	0
	Pancreatic cancer AND MMP25	1
	Pancreatic cancer AND MT6-MMP	1
Others	Pancreatic cancer AND MMP12	7
	Pancreatic cancer AND Metalloelastase	4
	Pancreatic cancer AND MMP19	2
	Pancreatic cancer AND MMP20	1
	Pancreatic cancer AND MMP21	1
	Pancreatic cancer AND enamelysin	1
	Pancreatic cancer AND MMP22	0
	Pancreatic cancer AND MMP23	0
	Pancreatic cancer AND cysteine array	9
	Pancreatic cancer AND MMP27	0
	Pancreatic cancer AND MMP28	2
	Pancreatic cancer AND epilysin	0
